# Immunization with recombinant rabies virus expressing Interleukin-18 exhibits enhanced immunogenicity and protection in mice

**DOI:** 10.18632/oncotarget.21065

**Published:** 2017-09-16

**Authors:** Weiwei Gai, Wenwen Zheng, Chong Wang, Gary Wong, Yanyan Song, Xuexing Zheng

**Affiliations:** ^1^ College of Veterinary Medicine, Jilin University, Changchun, China; ^2^ School of Public Health, Shandong University, Jinan, China; ^3^ State Key Laboratory of Veterinary Biotechnology, Harbin Veterinary Research Institute, Chinese Academy of Agricultural Sciences, Harbin, China; ^4^ CAS Key Laboratory of Pathogenic Microbiology and Immunology, Institute of Microbiology, Chinese Academy of Sciences, Beijing, China

**Keywords:** rabies virus, IL-18, immune responses, vaccine, antibody titer

## Abstract

Several studies have shown that interleukin-18 (IL-18) plays an important role in both innate and adaptive immune responses. In this study, we investigated the pathogenicity and immunogenicity of recombinant rabies virus expressing IL-18 (rHEP-IL18). Experimental results showed that Institute of Cancer Research (ICR) mice that received a single intramuscular immunization with rHEP-IL18 elicited the highest titers of serum neutralizing antibodies and the strongest cell-mediated immune responses to prevent the development of rabies disease, compared with immunization with the parent virus HEP-Flury. Mice inoculated with rHEP-IL18 developed significantly higher IFN-γ responses, increased percentages of CD4^+^ and CD8^+^ T-lymphocytes compared to HEP-Flury. Flow cytometry results show that rHEP-IL18 recruited more activated T- and B-cells in lymph nodes or peripheral blood, which is beneficial for virus clearance in the early stages of infection. A higher percentage of mice immunized with rHEP-IL18 survived wild-type rabies virus (RABV) challenge, compared to HEP-Flury mice. Our results show that rHEP-IL18 is promising as a novel vaccine for RABV prevention and control.

## INTRODUCTION

Rabies is a fatal zoonotic infectious disease caused by rabies virus (RABV) and afflicts nearly all mammalian hosts [1, 2]. The main feature of RABV is neuroinvasiveness, which refers to the ability to invade the central nervous system (CNS) from peripheral sites. Once clinical signs are present in infected humans or animals, rabies is almost 100% fatal [3]. According to the World Health Organization, there are approximately 55,000 human deaths each year worldwide, including 3000 cases in China [4–6]. Dogs and cats are the major reservoir of rabies and most human cases occur by bites from rabid dogs in developing countries in Africa and Asia [7–9]. Vaccination is the most effective way to prevent and control rabies in animals and humans [10–12]. In contrast to other RABV vaccines, most live-attenuated vaccines replicate very quickly and express large amounts of glycoprotein (G), thereby inducing strong adaptive immune responses that result in virus clearance [13]. Live-attenuated RABV vaccines and recombinant live vaccines have already been licensed, such as a recombinant vaccinia virus expressing the RABV G protein (VRG) and a live avirulent RABV, SAG-2, particularly for wild animals [14–17]. These vaccines provide effective immunization with a single dose and has practical, economical, and logistical advantages over conventional multi-dose vaccines with respect to the goal of eradicating rabies worldwide [16, 18–21]. In addition, live-attenuated RABV vaccines can induce immune responses to clear virulent RABV from the CNS and there is the possibility that such vaccines could serve as the foundation for the treatment of early stage human rabies infections [22, 23].

Previous studies show that interlukin (IL)-18 is a pleiotropic cytokine that plays an important role in both innate and adaptive immunity, with an ability to produce high amounts of IFN-γ [24, 25]. Its function is mainly reflected in the enhancement of T cell-mediated immunity [26], but IL-18 is a unique cytokine that can enhance innate immunity and both Th1- and Th2-driven immune responses [27, 28]. IL-18 enhances T cell-activated monocytes to induce IFN-γ production, which is dependent on the activation of intracellular NK-κB and PI3-kinase pathways. But IL-18, without help from IL-12, induces Th2 cells by induction of IL-4 production from naive T cells. Thus, IL-18 has the potential to stimulate both Th1 and Th2 responses [28]. Therefore, IL-18 is an interesting candidate for the modulation of immune responses.

In this study, the gene for IL-18 was cloned into the HEP-Flury cDNA clone. The recombinant RABV, rHEP-IL18, was rescued and used to immunize mice in comparison with HEP-Flury. Our results show that rHEP-IL18 is less pathogenic and more immunogenic compared to HEP-Flury in mice, with recruitment and activation of higher numbers of T and B cells, and provided enhanced protection against challenge with wild-type RABV.

## RESULTS

### Generation and characterization of recombinant attenuated RABV expressing murine IL-18 (rHEP-IL18) *in vitro*

**Figure 1:**
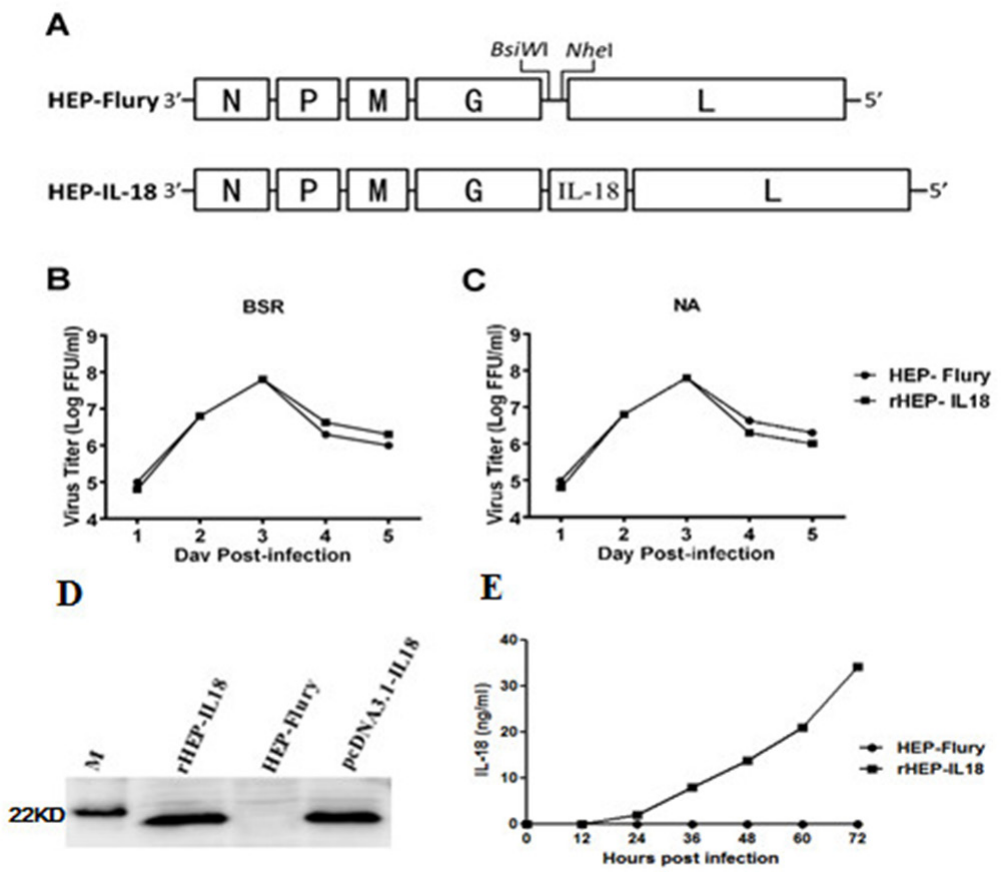
Construction and characterization of recombinant RABV expressing IL-18 *in vitro* **(A)** Schematic diagram for the construction of rHEP-IL18. The HEP-Flury vector was constructed from HEP-Flury strain by adding BsiWI and NheI sites between the G and L genes. Mouse IL-18 genes were cloned between the G and L. **(B)** Virus growth curves in BSR cells (B) and NA cells **(C)**. Cells were infected with HEP-Flury or rHEP-IL18 at a multiplicity of infection (MOI) of 0.01. Viruses were harvested at 1, 2, 3, 4, 5 and 6 dpi, and viral titers were determined as described in Materials and Methods. All titrations were carried out in quadruplicate. **(D)** The expression of protein IL-18 was determined by Western blotting analysis. The cells were collected and lysed for Western blotting after BSR cells were infected with rHEP-IL18 or HEP-Flury for 48h. Recombinant pcDNA3.1-IL-18 was used as a positive control. **(E)** The expression level of murine IL18 was determined by a commercial ELISA kit. Briefly, BSR cells were infected with rHEP-IL18 or HEP-Flury (MOI=1, 0.1, 0.01, or 0.001) for 48h, and the culture supernatants were harvested for measurement of murine IL18, each value was expressed as mean ±SD from three independent experiments.

The murine IL-18 gene was cloned into the HEP-Flury genome, as shown in Figure 1A. The insertion of the IL-18 gene was confirmed by sequencing the infectious clone and the recombinant virus, designated as rHEP-IL18, was rescued in BSR cells. In order to characterize rHEP-IL18 *in vitro*, viral growth kinetics were examined in BSR and NA cells. As shown in Figure 1B and 1C, a significant difference in values was not observed between recombinant viruses and the parent virus HEP-Flury, indicating that viral replication was not affected by the insertion of IL-18 gene. Moreover, the expression of IL-18 was detected by Western blotting. As shown in Figure 1D, one target band with molecular weights of 20 kDa corresponding to the positive control was detected, demonstrating that mouse IL-18 were expressed in the rHEP-IL18 infected cells, while no IL-18 was expressed in cells mock-infected or infected with HEP-Flury. In addition, the expression of IL-18 was measured by ELISA. As shown in Figure 1E, murine IL-18 was expressed in a dose-dependent manner in BSR cells infected with rHEP-IL18.

### Safety of rHEP-IL18 in mice

**Figure 2:**
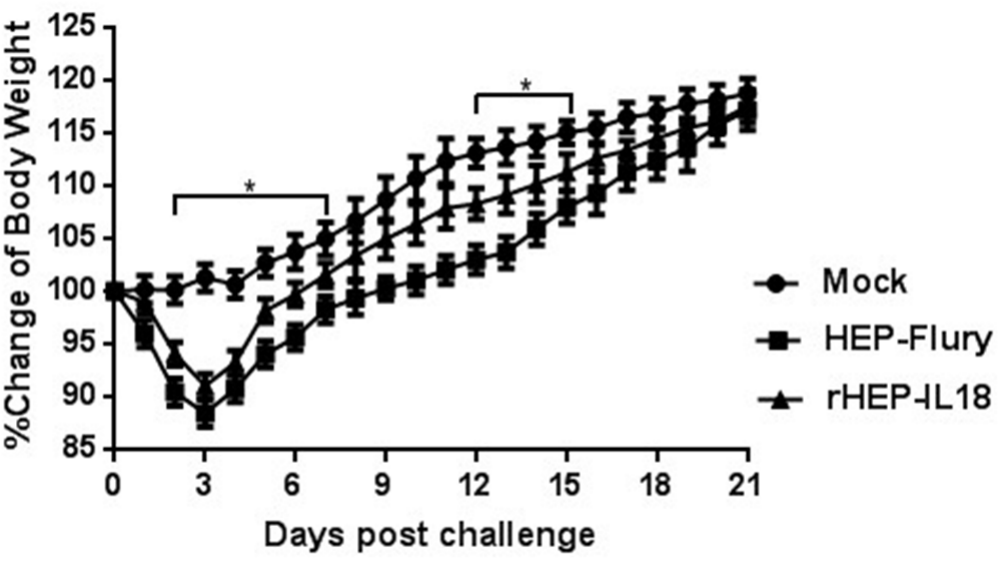
Pathogenicity of rHEP-IL18 in mice Groups of 10 ICR mice (6 to 8 weeks old) were infected intracranially with 10^6^ FFU of HEP-Flury, rHEP-IL18 virus or DMEM alone. Their body weights were monitored daily for 21 days. Representative data from all 10 mice in each group and are shown as mean values±SD.

As shown in Figure 2, mice injected with 1 × 10^6^ FFU of rHEP-IL18 lost less body weight than those injected with the same dose of HEP-Flury. Most of the mice regained their pre-infection body weight by 21 days post-infection (dpi). The data suggest that expression of IL-18 substantially decreased the pathogenicity of the live-attenuated RABV vaccine. No clinical symptoms were observed in all mice, such as abnormal behaviors or any neurological signs.

Neurovirulence was also evaluated for the vaccine candidates by i.c. route in suckling mice. One out of ten suckling mice injected via i.c. with 1 × 10^5^ FFU of HEP-Flury died. In contrast, inoculation of suckling mice via i.c. with rHEP-IL18 or DMEM resulted in no clinical signs or lethality. These results suggest that rHEP-IL18 may have an increased safety profile versus HEP-Flury *in vivo*.

### Immunogenicity of rHEP-IL18 in mice

**Figure 3:**
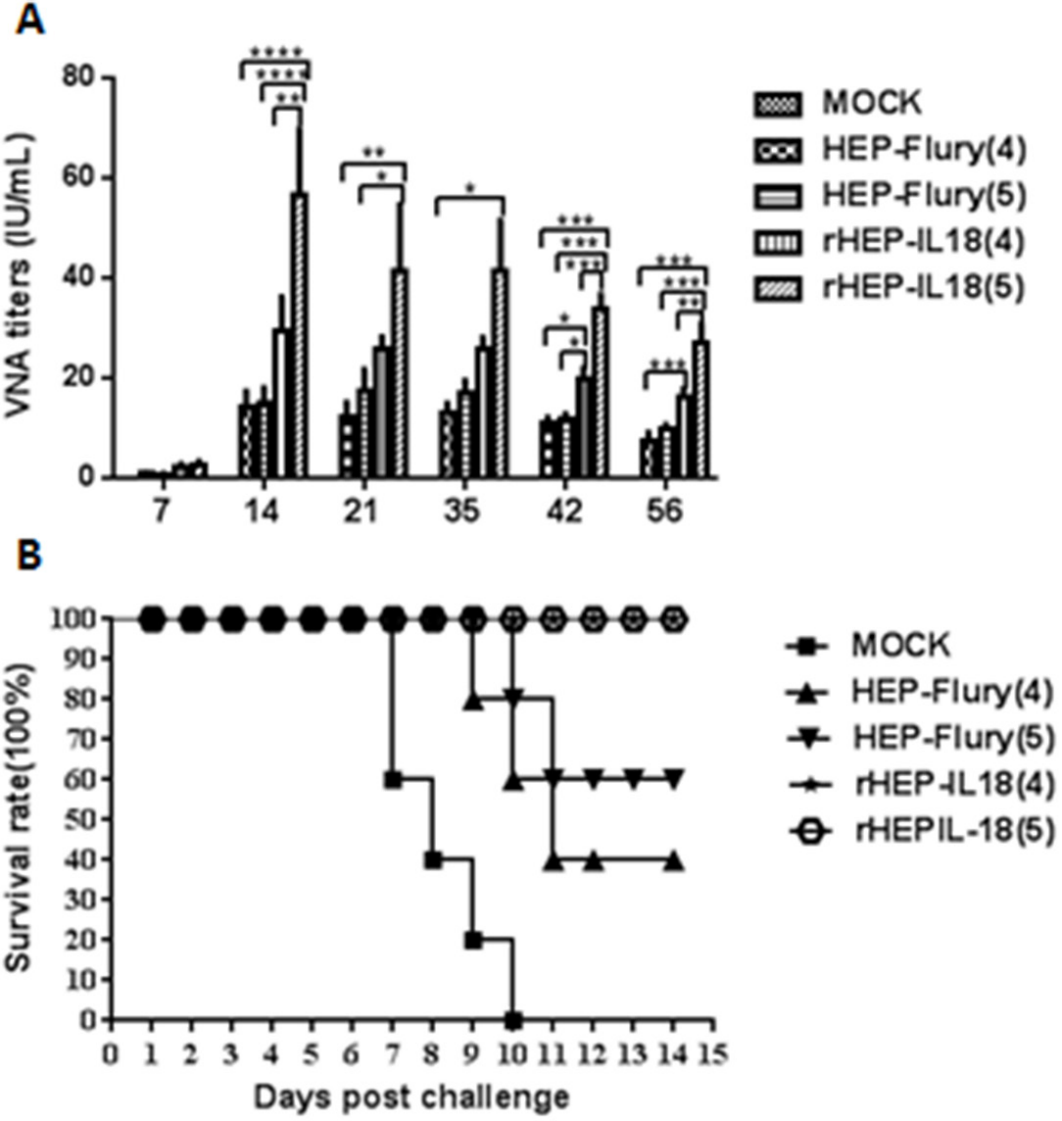
VNA titers and protection of rHEP-IL18 in mice **(A)** Groups of 10 ICR mice (n = 10) were respectively immunized with 1 × 10^4^ or 1 × 10^5^ FFU of rHEP-IL18, HEP-Flury or DMEM by the i.m. route. At days 7, 14, 21, 35, 42 and 56 after immunization, the peripheral blood was collected for VNA test. Titers were normalized to IU using the WHO standard. Data are analyzed by one-way ANOVA and asterisks indicate significant differences among the experimental groups (*p < 0.05; **p < 0.01; ***p < 0.001; ****p < 0.001). **(B)** Then at days 21 after immunization, mice were challenged with 10 × IMLD_50_ of street rabies virus strain HuNPB3 by the i.m. route and observed daily for 14 days, and survivorship was recorded.

Mice (n = 10) were immunized once via the i.m. route with either 1 × 10^4^ or 1 × 10^5^ FFU per mouse of rHEP-IL18 or HEP-Flury. Mice given DMEM were included as controls. Blood samples were collected at 7, 14, 21, 35, 42 and 56 dpi, and used for the VNA test. As shown in Figure 3A, the level of VNA in mice immunized with 1 × 10^4^ or 1 × 10^5^ FFU rHEP-IL18 (2.26 IU or 2.52 IU, respectively) were higher than that in those induced by immunization with HEP-Flury (< 0.85 IU) at 7 dpi. Significantly higher VNA titers were detected in mice immunized with 1 × 10^5^ FFU of rHEP-IL18 viruses at 14 dpi (56.78 IU) (P < 0.0001) and 21 dpi (41.24 IU) (P < 0.01) than mice immunized with HEP-Flury. Mice immunized with 1 × 10^4^ FFU of rHEP-IL18 induced higher level of VNA than HEP-Flury, but this difference was not statistically significant. VNA titers induced at 35 dpi was similar with that at 21 dpi, however, VNA titers decreased gradually from after 42 dpi to 56 dpi. Significantly higher VNA titers were still detected in mice immunized with 1 × 10^5^ FFU of rHEP-IL18 viruses at 42 dpi (33.91 IU) (P < 0.01) and 56 dpi (27.16 IU) (P < 0.01) than mice immunized with HEP-Flury. No VNA was detected in the DMEM group. Overall, the level of VNA observed is dependent on the vaccination dose.

To determine if higher VNA titers correlate with better protection, mice were challenged with 100 x IMLD_50_ of street rabies virus strain HuNPB3 in the muscle of the forelimb on day 21 after vaccination and observed for development of RABV disease and death for 14 days. As shown in Figure 3B, a higher proportion of survivors were observed among mice immunized with rHEP-IL18 than among those immunized with HEP-Flury. Immunization with 1 × 10^4^ FFU or 1 × 10^5^ FFU of rHEP-IL18 protected 100% of the mice, whereas immunization with 1 × 10^4^ or 1 × 10^5^ FFU of HEP-Flury provided only 40% or 60% protection, respectively. Together, the data indicate that rHEP-IL18 stimulated higher VNA responses and provided better protection compared to HEP-Flury.

### Recruitment of B and T cells *in vivo*

**Figure 4:**
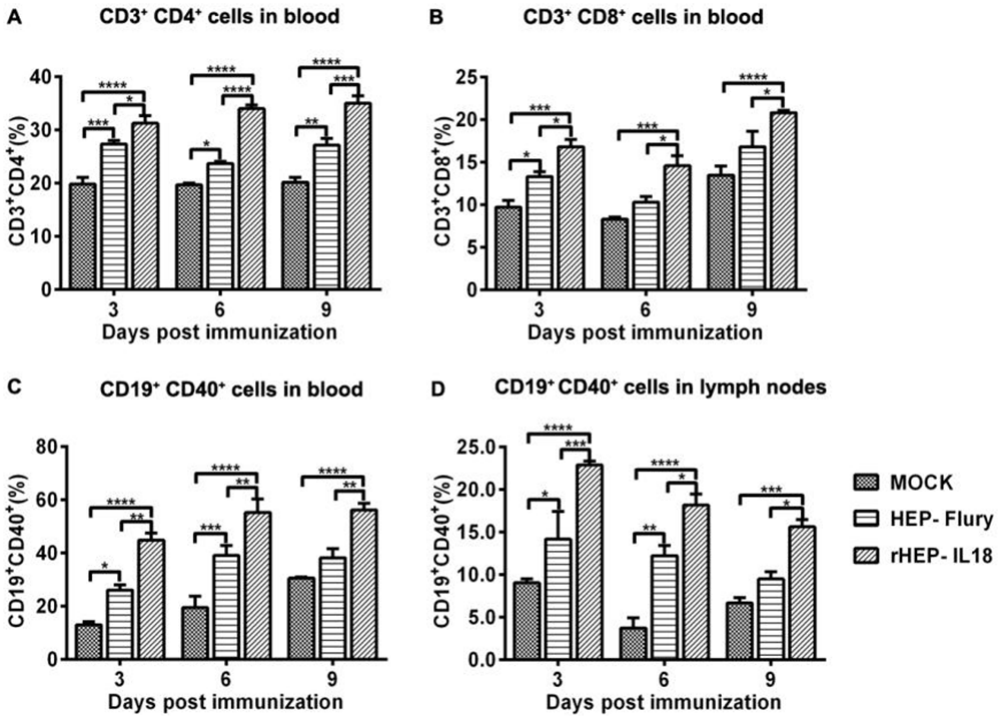
Recruitment and/or activation of T and B cells in blood and lymph nodes after rHEP-IL18 infection ICR mice were immunized i.m. with 1 × 10^5^ FFU of rHEP-IL18, HEP-Flury or DMEM. Blood and inguinal lymph nodes were collected from 3 mice per group at 3, 6, and 9 dpi. Single cell suspensions were prepared and stained with antibodies for T (CD3^+^, CD4^+^ and CD8^+^) from the blood **(A** and **B)** and B cells (CD19^+^ and CD40^+^) from the blood **(C)** and the lymph nodes **(D)**. Asterisks indicate significant differences among the experimental groups as analyzed by one-way ANOVA (*P < 0.05;**P < 0.01;***P < 0.001;****P < 0.0001).

To determine whether the expression of IL-18 results in the recruitment of higher numbers of B and T cells *in vivo*, flow cytometry was performed to quantify the immune cells in the peripheral blood and lymph nodes (CD3^+^, CD4^+^ and CD8^+^ for T cells and CD19^+^ and CD40^+^ for B cells) at 3, 6, and 9 dpi. Each group of mice was immunized once via i.m. with 1 × 10^5^ FFU per mouse of rHEP-IL18 or HEP-Flury. As shown in Figure 4A and 4B, significantly more CD4^+^ T cells and CD8^+^ T cells were detected in the blood of mice immunized with rHEP-IL18 than in those immunized with HEP-Flury or mock mice at 3, 6, 9 dpi. Figure 4C, 4D show that significantly more activated B cells (CD40^+^ and CD19^+^) were detected in blood and in lymph nodes of mice immunized with rHEP-IL18 than in mice immunized with HEP-Flury or DMEM at 6 and 9 dpi. The results indicate that rHEP-IL18 induced higher levels of T and B cell activation, and that more T and B cells were recruited and/or activated in lymph nodes and peripheral blood.

### Cell-mediated immune responses

**Figure 5:**
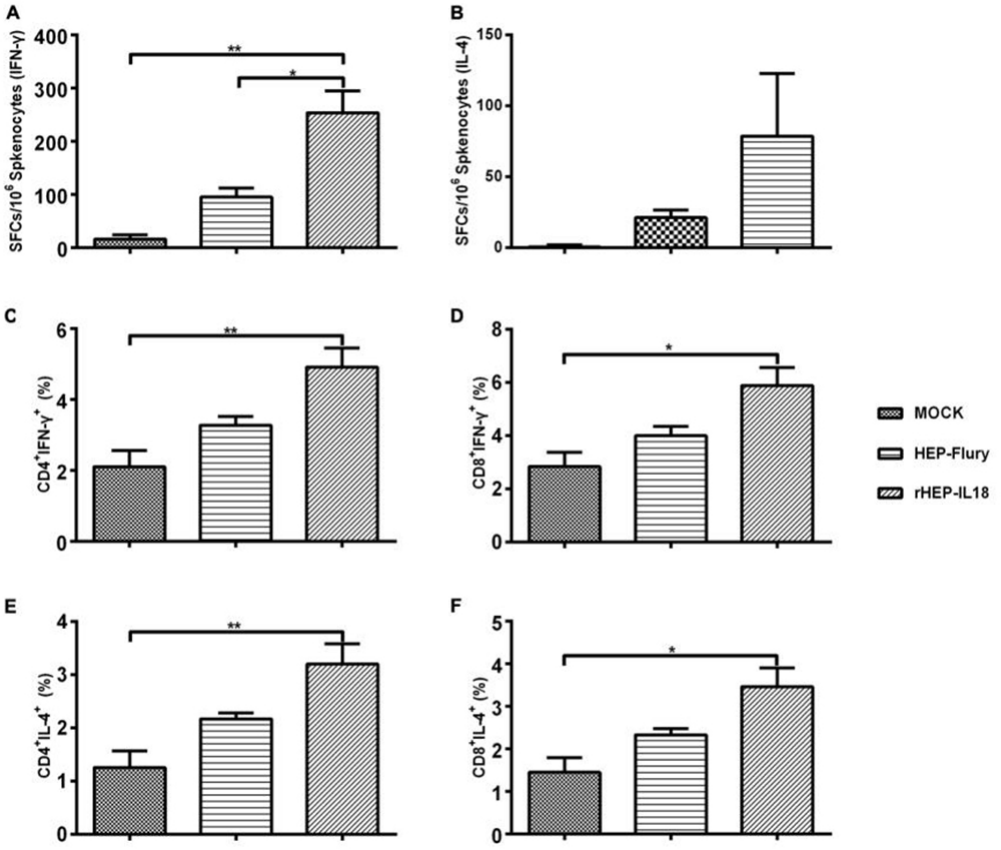
ELISpot analysis of IFN-γ and IL-4 secretion and ICS assays for antigen-specific CD4^+^ and CD8^+^ T cell secretion of IFN-γ and IL-4 in splenocytes The splenocytes were collected from 3 mice per group at 14 days after immunization, and splenocytes were assayed as described in Material and Methods. The secretion of IFN-γ **(A)** and IL-4 **(B)** were measured by a commercial ELISpot kit. Then the splenocytes were stained with mouse anti-CD4, -CD8, -IFN-γ, and -IL-4 monoclonal antibodies. The CD4^+^ T cells secreting IFN-γ **(C)** or IL-4 **(E)**, and CD8^+^ T cells secreting IFN-γ **(D)** or IL-4 **(F)** were shown. The data represent the means of subtraction values with SD and were analyzed by one-way ANOVA (*p < 0.05, **p < 0.01, ***p < 0.001).

RABV-specific IFN-γ and IL-4 expression were evaluated in splenocytes by ELISpot assays. As shown in Figure 5A, counts of IFN-γ expressing cells were significantly higher in mice immunized with rHEP-IL18 compared to HEP-Flury (p < 0.01) or DMEM-immunized mice (p < 0.05). As shown in Figure 5B, the rHEP-IL18 induced a higher level of IL-4, but this difference was not statistically significant. The results indicated that rHEP-IL18 elicited a higher Th1 like cellular immune response compared to other groups. The ability of rHEP-IL18 to induce IFN-γ- or IL-4-secreting CD4^+^ and CD8^+^ T cells were then measured. As shown in Figure 5C and 5D, rHEP-IL18 elicited higher numbers of IFN-γ- secreting CD4^+^ and CD8^+^ T cells, compared with the HEP-Flury and DMEM groups. As shown in Figure 5E and 5F, similar results were observed for IL-4-secreting CD4^+^ and CD8^+^ T cells. The data demonstrate that mice immunized with rHEP-IL18 elicited a notably enhanced IFN-γ- or IL-4-secreting CD4^+^ and CD8 ^+^ T cell response.

### IFN-γ, IL-2, IL-4 and IL-10 secretion from splenocytes of immunized mice

**Figure 6:**
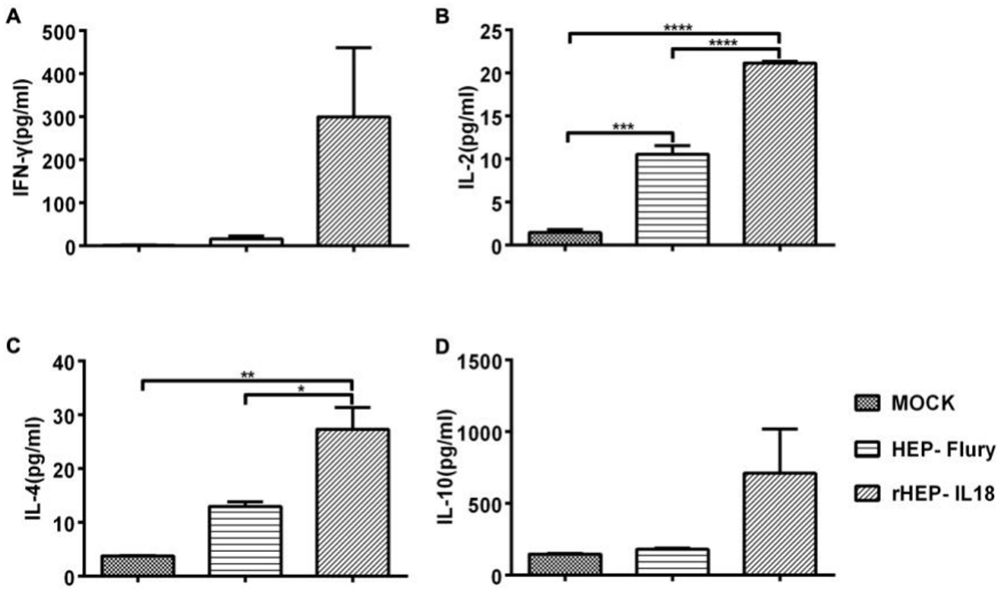
The quantities of IL-2, IL-4, IL-10 and IFN-γ secreted by splenocytes Splenocytes were isolated from 3 mice per group at 14 days after immunization, and cell-free supernatants were harvested at 48h after incubation and measured for the levels (pg/mL) of IFN-γ **(A)**, IL-2 **(B)**, IL-4 **(C)** and IL10 **(D)** by a commercial ELISA kit in duplicate. Representative data are shown as the mean ± SD of 3 mice per group and were analyzed using one-way ANOVA (*p < 0.05, **p < 0.01, ***p < 0.001).

Levels of secreted IFN-γ, IL-2, IL-4 and IL-10 from splenocytes were measured using commercial ELISA kits. As shown in Figure 6A and 6D, the levels of IFN-γ and IL-10 secreted in mice immunized with rHEP-IL18 were higher than those detected in the HEP-Flury or mock group. The levels of IL-2 secretion in mice immunized with rHEP-IL18 were significantly higher than those detected in the parent HEP-Flury (P < 0.0001) or DMEM treated mice (P < 0.0001) (Figure 6B). Similarly, the levels of IL-4 secretion in mice immunized with rHEP-IL18 were significantly higher than those detected in the parent HEP-Flury (P < 0.05) or DMEM treated mice (P < 0.01) (Figure 6C). The results indicate that rHEP-IL18 elicited enhanced Th1 and Th2 cellular immune responses.

## DISCUSSION

Previous studies have shown that the expression of cytokines/chemokines could further attenuate RABV virulence, induce relatively high titers of neutralizing antibodies, and enhance protective immune responses to wild-type RABV challenge [24, 29]. In order to improve the efficacy of RABV vaccines, several cytokines have been trialed as adjuvants to improve immune responses, including MIP-1α, MDC and GM-CSF [29, 30]. In this study, we selected IL-18 on the basis that this cytokines has been widely used as an adjuvant to enhance/regulate innate and adaptive immune responses of many vaccine antigens [31–33].

Our results show that the viral kinetics of rHEP-IL18 (up to 10^8^ FFU) was similar with those of the parent virus HEP-Flury in NA and BSR cells. ICR mice are a kind of out-breed mice, which own strong viability and adaptability, are commonly emplyed for the immunization experiment due to the high repeatability. The pathogenicity and immunogenicity of rHEP-IL18 was then investigated in ICR mice. Although weight loss occurred in mice vaccinated with rHEP-IL18 or HEP-Flury, no overt clinical symptoms, such as abnormal behavior or neurological signs, were observed. However, mice infected with rHEP-IL18 regained their original body weight earlier than mice infected with HEP-Flury, suggesting that IL-18 expression reduced the pathogenicity of the recombinant RABV vaccine.

Our results also showed that rHEP-IL18 induced higher VNA titers and provided better protection than HEP-Flury. Neutralizing antibody is a major component of humoral immunity contributing to protection against RABV infection, and plays an important role in the prevention or reduction of viral spread [34, 35]. Cell-mediated immunity has been proposed to play an important role against RABV infection and may be involved in viral clearance from the CNS [36]. However, it is not known whether IL-18 substantially impacts B cell responses. A previous study has reported that IL-18 has an effect on CD40^+^ B cell activation [37, 38]. In this study, mice immunized with rHEP-IL18 induced higher titers of VNA at earlier times compared to HEP-Flury at 7 dpi, increasing in titer when tested at 14 and 21 dpi. VNA titers induced at 35 dpi was similar with that at 21 dpi, and decreased gradually from 42 dpi. Furthermore, our data showed that IL-18 stimulated the maturation, activation, and/or recruitment of more CD19^+^ CD40^+^ B cells in lymph nodes and blood among immunized mice than HEP-Flury at 3, 6 and 9 dpi, which may contribute to the higher VNA induced than the parental virus.

IL-18 has a potent ability to produce IFN-γ, which activates immune cells either independently or with IL-12 [39]. The Th1 immune response is activated by IL-2 and IFN-γ [40, 41], whereas the Th2 immune response is activated by IL-4 and IL-10 [42]. Our data showed that rHEP-IL18 enhanced cytokines secretion of IFN-γ and IL-10, and had a significant effect on IL-2 and IL-4 cytokines secretion, indicating that rHEP-IL18 mediated/boosted immune responses via both the Th1 and Th2 pathways. Also, rHEP-IL18 induced a stronger and faster CD4^+^ and CD8^+^ T cell response in immunized mice *in vivo* than HEP-Flury, with higher numbers of peripheral CD4^+^ and CD8^+^ cells observed at 3, 6 and 9 dpi. The combination of the humoural and cell-mediated immune response data suggests a stronger adaptive immune response contributed to enhanced protection in rHEP-IL18-vaccinated mice.

Overall, these results demonstrated that IL-18 possesses strong adjuvant activity and that rHEP-IL18 enhanced humoral and cellular immune responses leading to enhanced protection. Therefore, the rHEP-IL18 is an improvement on the HEP-Flury vaccine and should be further characterized in a higher-order animal model.

## MATERIALS AND METHODS

### Ethics statement

Animal studies were carried out in strict accordance with prior approval from the Animal Welfare and Ethics Committee of School of Public Health, Shandong University (permit number SCXK-20150015). The environment and housing facilities used in this experiment satisfies the guidelines put forth by the National Standards of Laboratory Animal-Requirements of Environment and Housing Facilities (GB 14925-2001) of China. All efforts were made to minimize animal suffering.

### Cells, viruses, antibodies, and animals

BSR cells, a cloned cell line derived from BHK-21 cells, and mouse neuroblastoma (NA) cells were maintained in Dulbecco's modified Eagle's medium (DMEM) (Gibco, Invitrogen, China) supplemented with 10% fetal bovine serum (FBS) (Gibco, Grand Island, NY, USA). The RABV HEP-Flury reverse genetics system is provided by Professor Xiaofeng Guo (South China Agricultural University). Standard attack rabies virus strain CVS-11 and street rabies virus strain HuNPB3 were propagated in NA cells. Fluorescein isothiocyanate (FITC)-conjugated antibody against the RABV N protein was purchased commercially from Fujirebio Diagnostics, Inc. (Malvern, PA, USA). Anti-IL-18 monoclonal antibody was purchased from Santa Cruz Biotechnology, Inc (Santa Cruz, CA, USA). Antibodies used for flow cytometry analysis, such as anti-CD3, anti-CD4, anti-CD8, anti-CD19 and anti-CD40 were purchased commercially (BD Biosciences, USA). Female ICR mice (6-8 weeks old) were purchased from the Shandong University Laboratory Animal Center (Jinan, China).

### Construction of recombinant RABV cDNA clones

The vector HEP-Flury was digested with *BsiWI* and *NheI* between the G and RNA-dependent RNA polymerase (L) genes. Murine IL-18 was amplified from the mouse genome and inserted between the G and L genes to obtain HEP-Flury-IL18.

### Rescue of rRABV HEP-IL18

rHEP-IL18 was rescued as described previously [43, 44]. Briefly, 1 × 10^6^ BSR cells per well were grown overnight to 60%–80% confluence in six-well plates (Corning, Steuben County, NY, USA) in DMEM supplemented with 10% FBS. The cells were transfected with 2.0 μg of the full-length clone, 0.5 μg of N helper plasmid, 0.25 μg of P helper plasmid, 0.1 μg of L helper plasmid and 0.15 μg of G helper plasmid using the SuperFect transfection reagent (Qiagen, Hilden, Germany) according to the manufacturer instructions. After incubation at 37°C for 4 days, the culture medium was harvested and the cells were tested for rescued virus using FITC-conjugated anti-RABV N antibody (Centocor, Malvern, PA, USA). Fluorescence in the cells was measured with a fluorescence microscope. The rescued virus rHEP-IL18 strain was inoculated in NA cells and BSR cells for virus stock preparation and further experiments.

### Virus titration

Virus titration was performed using the direct fluorescent antibody assay in NA cells. In a 96-well plate, NA cells were inoculated with serial 10-fold dilutions of virus and incubated at 37 °C for 48 h. The culture supernatant was removed, and the cells were fixed with ice-cold 80% acetone for 30 min. The cells were washed twice with PBS and then stained with FITC-conjugated anti-RABV N antibody at 37 °C. Antigen-positive foci were counted under a fluorescence microscope and virus titer was calculated as fluorescent focus units per milliliter (FFU/mL). All titrations were carried out in quadruplicate.

### Multistep growth assays

Monolayer cultures of NA or BSR cell grown in a T25 cell culture flask were infected with HEP-Flury or rHEP-IL18 at an MOI of 0.01. After inoculation for 1h at 37 °C, the supernatant were removed and then the cells were washed twice with PBS (pH7.4). The NA or BSR cells were supplied with fresh growth medium containing 2% FBS and incubated in a 5% CO_2_ incubator at 37 °C. Samples of culture medium were harvested from the NA or BSR cells at 24, 48, 72, 96, 120 and 144 h post-infection for virus titration. Virus titer was determined in BSR cells using the direct fluorescent antibody as previously described.

### Western blotting

BSR cells were infected with different viruses at an MOI of 0.01 and incubated at 37 °C for 48 h and lysed with RIPA buffer (Thermo Scientific, USA). The lysates were centrifuged at 12,000 ×g for 15min and the supernatants were mixed with 5 × loading Buffer. IL-18 expression was analyzed by SDS-PAGE and transferred onto a fluoride (PVDF) membrane (Immobilin-P, Millipore, USA) for Western blotting with anti-IL-18 monoclonal antibody (1:500), followed by detection with HRP-conjugated goat anti-mouse IgG polyclonal antibody (1:20,000) and enhanced chemiluminescence.

### Safety studies

To determine whether rHEP-IL18 vaccination is safe in mice, 3 groups of 10 female ICR mice (6 to 8 weeks old) were administered 1 × 10^6^ FFU of rHEP-IL18, HEP-Flury or DMEM via the intracerebral (i.c.) route. Body weights were monitored daily for 21 days. Data were obtained from 5 mice in each group and presented as the mean value ± standard deviation (SD).

For neurovirulence analyses, 3 groups of 10 ICR suckling mice (5-day-old) were administered 1 × 10^6^ FFU of rHEP-IL18, HEP-Flury or DMEM via the i.c. route. Suckling mice were monitored daily for encephalitis and the numbers of surviving mice were recorded daily.

### Immunogenicity and protection studies

Female ICR mice (6-8 weeks old) were randomly divided into 5 groups and vaccinated intramuscularly (i.m.) in the hind legs with HEP-Flury or rHEP-IL18 at a concentration of 10^4^ or 10^5^ FFU. A control group was mock-vaccinated with DMEM at the same time points. At 21 days after immunization, mice were challenged i.m. with 100 × median intramuscular mouse lethal dose (IMLD_50_) of street rabies virus strain HuNPB3 in the muscle of the forelimb, and survival/body weights were observed daily for 21 days. During the observation period, any mice that developed clinical signs of rabies were humanely euthanized by cervical dislocation under isoflurane anesthesia.

### Fluorescent antibody virus neutralization tests (FAVN)

Blood was collected from mice 7, 14, 21, 35, 42 and 56 days after immunization by retro-orbital plexus puncture for measurement of VNA using a fluorescent antibody virus neutralization (FAVN) test as described previously [45]. Titers were calculated according to the Spearman and Kärber method [46].

### Flow cytometry

To investigate the effects of IL-18 expression on the recruitment of T and B cells in the peripheral blood and in lymph nodes, flow cytometry was performed using a LSR-II flow cytometer (BD Bioscience). Briefly, 10 female ICR mice (6-8 weeks) per group were inoculated i.m. with 1 × 10^5^ FFU of HEP-Flury, rHEP-IL18 or with medium alone. At days 3, 6 and 9 post-infection, the single cell suspension of peripheral blood or inguinal lymph nodes was harvested and stained with antibodies to T cell- (CD3^+^, CD4^+^, and CD8^+^) or B cell- (CD19^+^ and CD40^+^) markers (BD Biosciences Pharmingen, USA) for 30 min at 4 °C. A minimum of 20,000 events were counted.

### ELISpot assays

Splenocytes from vaccinated mice were isolated at 14 days after immunization and stimulated with inactivated HuNPB3 at a concentration of 10 μg/mL for 36 h at 37 °C. Secreted IFN-γ or IL-4 was quantified by the mouse IFN-γ/IL-4 ELISPOT kit (Mabtech AB, Stockholm, Sweden) according to manufacturer instructions. Spot forming cells (SFCs) were counted using an automated ELISpot reader (AID ELISPOT reader-iSpot, AID GmbH, GER).

### Intracellular cytokine staining (ICS)

Splenocytes were collected from 3 mice from each group at 14 days after immunization and were stimulated with inactivated HuNPB3 (10 μg/mL) with a protein transport inhibitor (BD Biosciences, Franklin, TN, USA). At 6 h post-stimulation, single cell suspensions were stained with anti-CD4 and anti-CD8 monoclonal antibodies (BD Biosciences, Franklin, TN, USA) for 30 min at 4 °C, as well as anti-IFN-γ and anti-IL-4 monoclonal antibodies (BD Biosciences, Franklin, TN, USA) for 30 min at 4 °C. All labeled cells were washed twice with PBS and 20,000 cells were analyzed in a LSR-II flow cytometer (BD Bioscience, Franklin, TN, USA).

### IL-2, IL-4, IL-10 and IFN-γ expression analysis using ELISA

Splenocytes were harvested from 3 mice of each group at 14 days after vaccination. The splenocytes (2 × 10^6^ cells/mL) were suspended in RPMI 1640 medium containing 10% FBS, and stimulated with inactivated HuNPB3 (10 μg/mL) at 37 °C for 48 h. The culture supernatants were then harvested and determined using an ELISA kit (Mouse IL-2, IL-4, IL-10 and IFN-γ ELISA kit, Mabtech AB). All assays were performed according to manufacturer instructions.

### Statistical analysis

The data were expressed as the mean ±SD and analyzed using GraphPad Prism 6 to determine statistically significant differences in the generated data by one-way analysis of variance (ANOVA). P < 0.05 was considered a statistically significant difference. *P <0.05;**P < 0.01; ***P < 0.001;****P < 0.0001.
